# Longitudinal reduction in fluoroscopy with continued use of 3-dimensional electroanatomic mapping systems in catheter ablation of supraventricular tachycardia – then and now

**DOI:** 10.1016/j.ipej.2024.06.010

**Published:** 2024-06-29

**Authors:** Yi Yi Chua, Julian Cheong Kiat Tay, Eric Tien Siang Lim, Xuanming Pung, Daniel Thuan Tee Chong, Kah Leng Ho, Chi Keong Ching

**Affiliations:** Department of Cardiology, National Heart Centre Singapore, Singapore

**Keywords:** Supraventricular tachycardia, Ablation, Fluoroscopy time, Electroanatomic mapping

## Abstract

**Background:**

Catheter ablation is a first-line treatment for symptomatic, recurrent supraventricular tachycardia (SVT). This study aims to demonstrate if 3D-electroanatomic mapping (EAM) during SVT ablation reduces fluoroscopy time (FT) and determine if further reductions in FT are observed longitudinally.

**Methods:**

All cases of SVT ablation between May 2011–May 2022 at a single tertiary centre were prospectively recruited. FT between the cohorts with and without EAM were compared. Within the EAM subset, the trend of FT across the years was analysed.

**Results:**

There were 1758 cases included, 563 without EAM, 1195 with EAM. EAM was associated with a longer procedure time (mean + 8.8 min, p = 0.001), but with mean reductions in FT and dose area product (DAP) by 19.6 min and 18 621 mGy*cm2 respectively (p < 0.001). There was comparable efficacy without any increase in complication rates. Over time (2011–2022), further reduction in FT of 0.9 min year on year was observed (p = 0.001). Between 2011 and 2017, there was a significant reduction in FT of 1.1 min year on year (p = 0.019), which was not observed from 2017 onwards (p = 0.061). The greatest reduction in FT was after the first year of adoption.

**Conclusion:**

EAM in SVT ablation reduces fluoroscopy use. FT was initially observed to reduce further over time before plateauing, likely due to increased operator experience. While there is increased interest in zero fluoroscopy SVT ablation, complementary use of fluoroscopy may still be necessary in complex cases.

## 1 Introduction

Supraventricular tachycardia (SVT) is a common tachyarrhythmia that can be associated with significant morbidity. While medical therapy with beta-blockers, calcium-channel blockers and other antiarrhythmic drugs may be considered for chronic therapy, catheter ablation is the treatment of choice for symptomatic patients given its high success and low complication rates [1].

Catheter ablation conventionally requires the use of fluoroscopy. However, fluoroscopy use exposes patients and staff to potentially harmful stochastic and deterministic effects of ionising radiation [2]. One hour of fluoroscopy is estimated to be associated with an increase in lifetime risk of fatal malignancy by 0.03 % for patients [2]. Thus, keeping radiation dose as low as reasonably achievable is of paramount importance.

The advent of 3D electroanatomic mapping (EAM) has led to a reduction in fluoroscopy use and consequently radiation exposure during SVT ablation, without compromising on short and long-term outcomes and complication rates [[3], [4], [5]]. However, little is known whether the degree of fluoroscopy reduction changes with time, and contemporary real-world data remains scarce.

The aims of this study are to provide contemporary data on the impact of EAM during SVT ablation on fluoroscopy time (FT) and determine if further reductions in FT are observed longitudinally. We hypothesise that over time, with continued use and wider adoption of EAM across all subtypes of SVT ablation, greater operator experience, and technological advancements in EAM, there will be further reductions in radiation exposure as estimated by FT.

## 2 Materials and methods

All cases of atrioventricular nodal re-entry tachycardia (AVNRT), atrioventricular re-entry tachycardia (AVRT), and/or atrial tachycardia (AT) ablation between May 2011 to May 2022 at the National Heart Centre Singapore, a tertiary centre, were prospectively recruited. Cases with concurrent ablation of ventricular arrhythmias, atrial fibrillation and atrial flutter were excluded.

All procedures were performed under intravenous sedation with midazolam and fentanyl as required. Local anaesthetic with lignocaine was administered at the vascular access sites, which were the femoral venous site for all patients and performed under ultrasound guidance. When access to the left side of the heart was required, this was achieved either using the retrograde aortic approach or transeptal approach with or without intra-cardiac echocardiography (ICE), according to the operator's preference. Fluoroscopy was performed using Philips FD10 (Philips Electronics North America, Andover, MA, USA). The minimum dose compatible with adequate imaging was used during the advancement of the catheters into the conventional locations and for confirmation of the catheter positions. Once suitable His signals were acquired, the area was tagged on the mapping screen. The CardioLab EP recording system (GE Healthcare, Cleveland, OH, USA) was utilised for all electrophysiology studies. Standard protocols and procedures, depending on the arrhythmic substrate, were used for all ablation procedures.

In this centre, EAM was first adopted in 2011. The decision for EAM use was operator-dependent and made before the procedure. The choice of EAM system, either EnSite™ Precision™ or X™ (St Jude Medical, St Paul, MN, USA), CARTO® 3D (Biosense Webster Inc, Diamond Bar, CA, USA), or Rhythmia (Boston Scientific, San Jose, CA, USA), was made according to operator's preference. In brief, the EnSite system uses impedance measurements between individual catheter electrodes and external patches placed on the chest to project a 3D image of the catheters, the CARTO system utilises ultralow intensity magnetic fields to triangulate the location and orientation of specialised catheters embedded with location sensors on its tip, while the Rhythmia system uses a hybrid tracking technology utilising both magnetic and impedance-based localisation features by using sensor coils and back patches. For all systems, three pairs of orthogonal skin patches were applied over the chest and on the back, in the usual recommended positions. Either the coronary sinus catheter or an external patch was selected as the reference.

Procedure time (in minutes) was defined as the time interval from the initial access site puncture to removal of all catheters. FT (in minutes) was defined as the cumulative duration of fluoroscopy during the entire procedure. Radiation dose (in mGy*cm2) was the calculated dose area product (DAP) for the patient.

Procedural success was defined based on type of SVT: (i) AVNRT - the absence of inducible tachycardia >20 min after the last radiofrequency catheter ablation (RFCA) application and the presence of up to a single atrioventricular nodal echo with intravenous isoprenaline challenge; (ii) AVRT - non-inducibility of tachycardia, loss of pre-excitation (if manifested) and/or loss of retrograde accessory pathway conduction after 20 min of observation following the last applied RFCA; (iii) AT — the inability to reinitiate the tachycardia despite intravenous isoprenaline challenge or programmed electrical stimulation down to atrial effective refractory period (ERP). We also categorised patients into single right or left-sided pathway ablations based on the cardiac chamber location of the final RFCA lesion that terminated the clinical tachycardia reliably and render it non-inducible. Patients with multiple pathways were categorised separately.

Statistical analysis was performed using SPSS version 29.0 (SPSS Inc, Chicago, IL, USA). Continuous variables were expressed as mean values with their associated standard deviations and compared using Student's t-test. Categorical variables were analysed using Fisher's exact test. Linear regression analysis was used to analyse FT over time.

## 3 Results

**Fig. 1:**
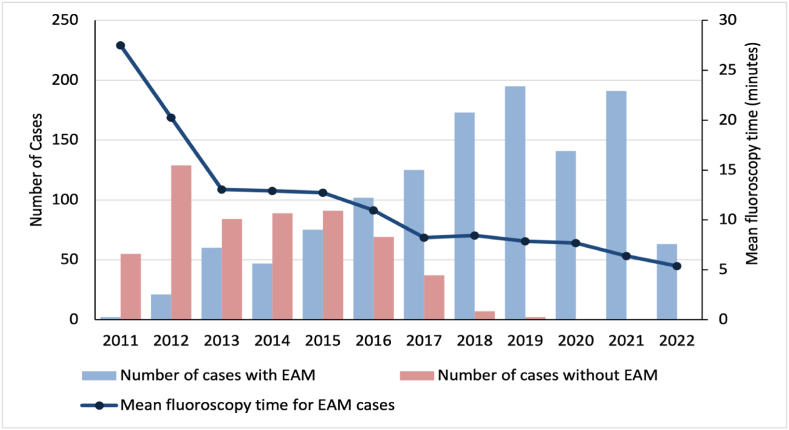
The clustered bar chart illustrates the number of cases of supraventricular tachycardia catheter ablation with and without electroanatomic mapping (EAM) from May 2011–May 2022. The dip in the number of cases using EAM in 2020 is likely related to COVID-19 pandemic. The superimposed line graph illustrates the mean fluoroscopy time (minutes) in cases of supraventricular tachycardia ablation with EAM from May 2011–May 2022.

Over this 11-year period, there were a total of 1758 cases of SVT ablation included in this study with a mean age of 44.0 ± 18.9 years, and approximately half were males (53.0 %). There were 124 paediatric cases (defined as aged 18 or less). Five hundred and sixty-three cases (32.0 %) were performed without EAM while the majority, 1195 cases (68 %) were with EAM. Among the patients in the non-EAM cohort, 321 (57.0 %) had AVNRT, 244 (43.3 %) had AVRT and 9 (1.6 %) had AT. Among the patients in the EAM cohort, 700 (58.6 %) had AVNRT, 513 (42.9 %) had AVRT and 38 (3.2 %) had AT. The distribution of EAM and non-EAM cases through 2011 to 2022 is shown in Fig. 1.

Comparison of baseline characteristics and procedural details between the EAM cohort and non-EAM cohort are shown in Table 1. Between the two cohorts, there were no significant differences (p > 0.05) in age, sex, history of prior ablation, history of structural heart disease and/or ischaemic heart disease, and diagnosis (AVNRT, AVRT, AT). Among the 117 cases with underlying structural heart disease and/or ischaemic heart disease, 16 cases have congenital heart disease.

**Table 1:** Comparison of baseline and procedural characteristics between the cases with and without electroanatomic mapping (EAM) during supraventricular tachycardia ablation.

	No. Of cases missing (%)	Without EAM (n = 563)	With EAM (n = 1195)	p-value
Age - year	0 (0)	43.1 ± 17.7	44.4 ± 19.4	0.156
Female sex - no. (%)	0 (0)	267 (47.2)	559 (46.8)	0.838
Prior ablation - no. (%)	0 (0)	30 (5.3)	81 (6.8)	0.293
Underlying structural heart disease and/or IHD - no. (%)	0 (0)	35 (6.2)	82 (6.9)	0.682
AVNRT - no. (%)	0 (0)	321 (57.0)	700 (58.6)	0.535
AVRT - no. (%)	0 (0)	244 (43.3)	513 (42.9)	0.877
AT - no. (%)	0 (0)	9 (1.6)	38 (3.2)	0.058
Single right-sided pathway ablated - no. (%)	0 (0)	375 (66.6)	814 (68.1)	0.548
Single left-sided pathway ablated - no. (%)	0 (0)	164 (29.1)	292 (24.4)	0.041
>1 pathway ablated during procedure - no. (%)	0 (0)	21 (3.7)	80 (6.7)	0.012
Use of stereotaxis - no. (%)	0 (0)	7 (1.2)	24 (2.0)	0.332
Use of intracardiac echocardiography - no. (%)	0 (0)	1 (0.2)	82 (6.9)	<0.001
Procedure time - minutes	252 (14.3)	97.6 ± 50.0	106.3 ± 48.7	0.001
Radiofrequency application time - seconds	343 (19.5)	364.5 ± 312.6	393.4 ± 387.0	0.147
Fluoroscopy time - minutes	240 (13.7)	28.7 ± 23.4	9.2 ± 13.3	<0.001
Fluoroscopy dose area product - mGy*cm [2]	230 (13.1)	24 887.3 ± 59 514.5	6266.3 ± 5292.4	<0.001
Immediate complications - no. (%)	0 (0)	1 (0.2)	7 (0.6)	0.449
Successful ablation - no. (%)	0 (0)	560 (99.5)	1186 (99.2)	0.431

AVNRT; atrioventricular nodal re-entry tachycardia, AVRT; atrioventricular re-entry tachycardia, AT; atrial tachycardia, IHD; ischaemic heart disease. Plus-minus values are means ± standard deviation.

Cases with EAM use was less likely to have single left-sided pathways ablated (p = 0.041) and more likely to have >1 pathway ablated during the procedure (p = 0.012). The use of stereotaxis did not differ significantly between the groups, though the cases with EAM use were more likely to use intracardiac echocardiography (ICE) (p < 0.001).

The mean procedure time was 106.3 ± 48.7 min in the EAM cohort and 97.6 ± 50.0 min for the non-EAM cohort. The use of EAM was associated with a longer procedure time (mean + 8.8 min, p = 0.001), but with mean reductions in FT and DAP by 19.6 min and 18 621.1 mGy*cm2 respectively (both p < 0.001). There were 131 zero-fluoroscopy cases. When categorised according to the number and site of pathway(s) ablated (single right-sided pathway, single left-sided pathway, >1 pathway ablated during procedure), we found that the difference in procedure time was mainly contributed by single right-sided pathway ablations, where the procedure time was significantly longer in the EAM cohort (100.1 ± 43.6 min) than the non-EAM cohort (87.4 ± 35.2 min). There was no significant difference between the procedure time for single left-sided pathway ablations and that for multiple pathways. Reductions in mean fluoroscopy time between the non-EAM cohort and EAM cohort were seen regardless of the number and site of pathway(s) ablated (Table 2). There was no difference in radiofrequency application time between the EAM and non-EAM cohorts (p = 0.147).

**Table 2:** Comparison of procedural characteristics between the cases of ablation of single right-sided pathway, single left-sided pathway, and multiple pathways.

	Single right-sided pathway			Single left-sided pathway			Multiple pathways		
	Without EAM	With EAM	p-value	Without EAM	With EAM	p-value	Without EAM	With EAM	p-value
Procedure time – minutes	87.4 ±35.2	100.1 ±43.6	<0.001	111.8 ±52.3	102.7 ±36.8	0.056	153.8 ±61.4	165.9 ±70.2	0.481
Fluoroscopy time – minutes	22.6 ±16.4	7.1 ±10.9	<0.001	38.3 ±26.6	11.8 ±11.6	<0.001	53.3 ±33.8	19.0 ±26.9	<0.001
Fluoroscopy dose area product – mGy*cm [2]	21 093.3 ± 65 928.6	4858.0 ± 11 744.0	<0.001	31 787.3 ± 43 969.4	8144.2 ± 13 022.8	<0.001	32 761.0 ± 26 032.8	13 137.4 ± 20 152.2	0.001

EAM; electroanatomic mapping. Plus-minus values are means ± standard deviation.

In terms of efficacy and safety, there were no significant differences between EAM and non-EAM cohort in terms of acute procedural success (p = 0.431) and complication rates (p = 0.449) as shown in Table 1. The complications described are those that occur intra-procedurally or immediately post-procedure. Of the 8 cases with complications, 3 were cases of cardiac tamponade requiring pericardiocentesis, 2 were cases of small pericardial effusions that were asymptomatic and did not require pericardiocentesis, 2 were cases of groin haematomas, and 1 was a case of ventricular fibrillation collapse during manipulation of the His catheter requiring cardioversion. All patients were discharged from the hospital uneventfully.

In the non-EAM cohort, cases with and without a history of prior ablation did not have significant differences between their procedure time (p = 0.570), radiofrequency ablation time (p = 0.401), and fluoroscopy time (p = 0.589). In the EAM cohort, cases with a history of prior ablation had significantly longer procedure time (p < 0.001), radiofrequency application time (p < 0.001), and fluoroscopy time (p = 0.012) than cases without a history of prior ablation. In both the non-EAM and EAM cohort, cases with a history of prior ablation were more likely to have AVRT and less likely to have AVNRT as compared with cases without a history of prior ablation (p < 0.05).

**Fig. 2:**
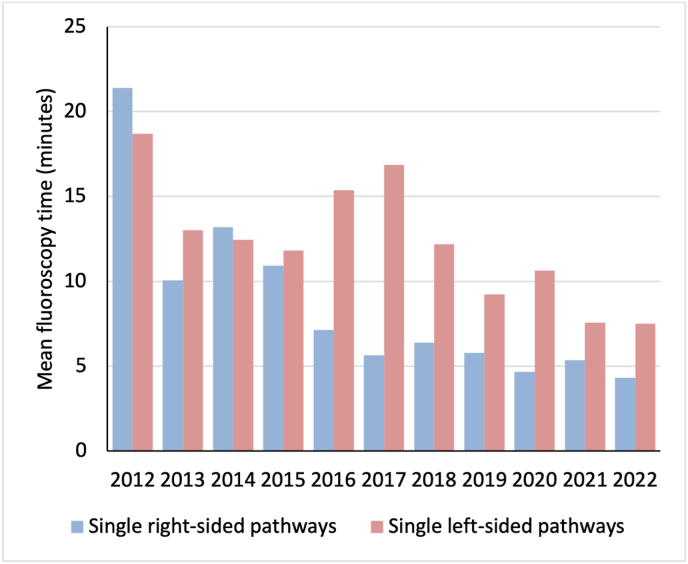
The clustered bar chart illustrates mean fluoroscopy time (minutes) for ablation with electroanatomic mapping, for single right-sided pathways and single left-sided pathways from 2012 to 2022.

As shown in Fig. 2, from 2011 to 2022 the mean fluoroscopy time for cases of single right-sided pathway ablations and single left-sided pathway ablations with EAM reduced over time, though this appears more apparent for the group including right-sided pathways.

Of the 1195 EAM cases, 908 cases (76.0 %) of the cases used EnSite, 260 cases (22.0 %) used CARTO, and 27 cases (2.2 %) used Rhythmia. There were statistically significant differences between the procedure time, radiofrequency application time, fluoroscopy time and dose area product (Table 2). Procedure time for cases using EnSite were significantly shorter those using CARTO (p < 0.001) and Rhythmia (p = 0.008) on post-hoc analysis. Radiofrequency application time in cases using CARTO was significantly longer than cases using EnSite (p = 0.031) but not between the other groups on post-hoc analysis. Fluoroscopy time for cases using EnSite were significantly shorter than those using CARTO (p = 0.27) but not significantly different from those using Rhythmia (p = 0.977). Fluoroscopy dose area product for cases using EnSite were also significantly less than those using CARTO (p = 0.001) but not significantly different from those using Rhythmia (p = 0.998) (see Table 3).

**Table 3:** Comparison between the different EAM systems – EnSite, CARTO and Rhythmia.

	EnSite (n = 908)	CARTO (n = 260)	Rhythmia (n = 27)	p-value
Procedure time – minutes	102.3 ±45.3	117.7 ±56.1	134.85 ±59.5	<0.001
Radiofrequency application time - seconds	373.8 ±378.9	453.4 ±414.8	486.9 ±317.3	0.023
Fluoroscopy time – minutes	8.57 ±13.0	11.3 ±14.4	9.18 ±10.1	0.036
Fluoroscopy dose area product – mGy*cm [2]	5445.11 ±11 490.2	9210.7 ±17 359.5	5622.6 ±9008.1	0.001

The trend of the mean fluoroscopy time for SVT ablation with EAM from 2011 to 2022 is shown in Fig. 1. After controlling for potential confounding factors including age, sex, use of stereotaxis, use of ICE, type and number of SVT, history of prior ablation, and structural heart disease and/or ischaemic heart disease, we found over time (2011–2022), further reduction in FT was observed, with a mean reduction of 0.9 min year on year (p < 0.001).

EAM was gradually adopted through the years with the number of cases per year plateauing after 2017. Between 2011 and 2017, there was a significant reduction in FT (mean of −1.1 min year on year, p = 0.019), which was not observed from 2017 onwards (p = 0.061). The greatest reduction in FT was observed after the first year of adoption, with a mean reduction of 7.2 min between 2012 and 2013. However, based on Fig. 2, there is a further non-significant trend for FT reduction when the fluoroscopy-only approach was completely phased out from 2020.

## 4 Discussion

The use of EAM has become common place and almost staple in most tertiary electrophysiology centres. EAM has allowed us to minimise fluoroscopy and the harmful effects of ionising radiation. In this study, we have described the patient and procedural characteristics in patients who undergo SVT ablation. Apart from exploring the differences between the EAM and non-EAM cohort, this study also highlights the changes in fluoroscopy use amongst EAM cases longitudinally over time.

Our study demonstrated that the use of EAM is associated with a significant reduction in fluoroscopy time (mean reduction of 19.6 min, p < 0.001) in SVT ablation, including cases of single right-sided pathway ablation, single left-sided pathway ablation, and that in multiple pathways. The use of EAM, however, was associated with a slightly longer procedure time of 8.8 min (p < 0.001). The longer procedure time may not be due to the use of EAM per se, but rather a reflection that more complex cases were more likely to be performed with EAM. Nonetheless, the marginally longer procedure time associated with EAM use is unlikely to have clinically significant impact on day-to-day practice given that the average procedure time in our study was 103 min. In our study, there were more cases of ablation of multiple pathways (p = 0.012) and single left-sided pathways (p = 0.041), as well as the concomitant use of ICE (p < 0.001) in our EAM cohort.

Some limitations exist. These difference in characteristics such as the number of cases of ablation of multiple pathways and single left-sided pathway, and use of ICE between the EAM and non-EAM cohorts may however lead to a selection bias. In addition, the non-EAM cases occurred between 2011 and 2019 with reducing numbers as time passed, whereas the EAM cases occurred between 2011 and 2022 with increasing numbers as time passed, which led to the comparison occurring between different time periods. Some cases were missing data on procedure time, radiofrequency application time, fluoroscopy time and dose area product, which reduced the power of the study and may have introduced bias.

While multiple studies have demonstrated that the use of EAM reduces fluoroscopy use in SVT ablation in both adult and paediatric population [3,6,7], there are no studies to date describing the change in fluoroscopy use with continued use of EAM over time. Our study demonstrated that over time, with increased use of EAM, fluoroscopy time was further reduced, and this was likely to reflect increased operator experience and procedural efficiency. The greatest reduction in fluoroscopy time was observed during the first year of adoption, with a mean reduction of 7.2 min. Further reduction in fluoroscopy time with EAM use plateaued after 6 years. Data on each individual operator's experiences in this study should ideally be taken into account, but is however lacking.

As shown in our study, the efficacy and safety of EAM use was comparable to conventional fluoroscopy-only SVT ablations. The acute procedural success was >99 % in both cohorts. Likewise, complication rates were low in both the non-EAM cohort (0.2 %) and the EAM cohort (0.6 %). Although data on long-term success and late complications are not tracked in this study, several studies have demonstrated that as compared with conventionally performed ablations under fluoroscopic guidance only, ablations performed with the use of EAM to minimise fluoroscopy has similar acute and long-term success rates without increase in complication rates [5,[7], [8], [9]].

There is increased interest in zero fluoroscopy SVT ablation with studies demonstrating significant reduction in cancer incidence and mortality [5,10]. In our study, there were 131 zero-fluoroscopy cases. However, complementary use of fluoroscopy may still be necessary in select cases such as those with multiple SVTs, complex congenital conditions, or distorted cardiac anatomy. In cases of left-sided SVT ablation, fluoroscopy may be helpful for safer left-sided access especially for those who undergo retrograde aortic approach. In our study, though EAM reduced the mean fluoroscopy time for cases of isolated left-sided pathway ablation and that for multiple pathways, the mean fluoroscopy time for these cases remained higher than that for isolated right-sided pathway ablation (Table 2). In complex cases, concomitant use of multimodality imaging such as pre-procedural cardiac computed tomography (CT) or magnetic resonance imaging (MRI) for 3D image integration as well as intraprocedural intracardiac echocardiography (ICE) have also been shown to reduce fluoroscopy time significantly [11]. [12].

Currently, routine use of 3D EAM system is still not standard practice. The Asia Pacific Heart Rhythm Society (APHRS) expert consensus gives class I recommendations for 3D EAM use only in patients who are pregnant, of paediatric age-group and those with mid-septal/para-Hisian pathways [13]. In this study, there were 124 paediatric cases (defined as aged 18 or less) and majority (70.2 %) used EAM. Despite this recommendation by APHRS, the benefits of EAM use extend beyond radiation exposure reduction. It allows better understanding of anatomy and pathophysiology of arrhythmia particularly in complex cases, facilitates training of EP fellows with continuous catheter visualisation and possibly reduction in orthopaedic-related risks with use of radiological protection measures in zero-fluoroscopy cases, all of which are intangible benefits that cannot be measured. Potentially complex cases include cases with congenital heart disease, where the use of EAM is associated with acute procedural success [14], likely through improved understanding of arrhythmia circuits and identification of ablation targets. In our study, there were only 16 cases with congenital heart disease of which 12 used EAM. The APHRS expert consensus also gives a class IIa recommendation for the use of 3D mapping system in redo ablation procedures, cases with impaired catheter stability, after catheter dislodgement, when consecutive mapping from different anatomical sites is performed, and for localisation of accessory pathways with lower success and higher recurrence rates [13]. In our EAM cohort, cases with a prior history of ablation had a significantly longer procedure time (p < 0.001), radiofrequency application time (p < 0.001), and fluoroscopy time (p = 0.012) than cases without a prior history of ablation, likely reflecting the more complex nature of these cases.

Previous studies reviewing the cost effectiveness of EAM use in catheter ablation have suggested that EAM use is cost effective in paediatric cases and for atrial fibrillation ablation, but not so in cases of SVT ablation [15,16]. In these studies however, the impact of fluoroscopy reduction on medical staff and that on non-fatal cancers in patients were not taken into account, potentially underestimating the benefits of EAM. Overall, the use of EAM should be guided by clinical need, affordability and anticipated cost effectiveness. Perhaps with further cost reduction, EAM may be used routinely in all ablation procedures to minimise the deleterious effects associated with fluoroscopy and improve ablation outcomes.

The three commercially available EAM systems are EnSite, CARTO and Rhythmia, all of which uses slightly different technologies and have their pros and cons [17]. Comparative cost, efficacy and safety between these three systems during SVT ablations have yet to be studied. In our study, the use of EnSite as compared to CARTO was associated with a shorter procedure time, radiofrequency application time, fluoroscopy time and dose area product, but not significantly different when compared to Rhythmia. However, the choice of EAM system was operator dependent and not randomized, which could have led to selection bias. More studies are required to draw comparison between these EAM systems.

## 5 Conclusions

In summary, the use of 3D electroanatomic mapping systems have allowed us to better adhere to the radiation usage principle of “as low as reasonably achievable” during SVT ablation. Our study provides contemporaneous evidence of EAM benefits in fluoroscopy time reduction and consequently radiation exposure with good efficacy and safety and suggests that EAM use comes with a learning curve.

## Data availability statement

Data is unavailable due to privacy or ethical restrictions.

## Funding statement

This research received no external funding.

## Ethics approval statement

Ethical review and approval were waived for this study due to use of anonymized data from another registry.

## Patient consent statement

Not applicable.

## Permission to reproduce material from other sources

Not applicable.

## Clinical trial registration

Not applicable.

## Author contributions

Both authors YY Chua and JCK Tay contributed equally and should be considered as co-first authors. YY Chua and JCK Tay were involved in literature review, data acquisition, statistical analysis, drafting of manuscript and final validation of manuscript. XM Pung, ETS Lim, DTT Chong, KL Ho and CK Ching were involved in drafting and final validation of manuscript.

## Declaration of competing interest

The authors declare that they have no known competing financial interests or personal relationships that could have appeared to influence the work reported in this paper.
